# Association between serum 25- hydroxyvitamin D and albuminuiria in middle-aged and older Chinese patients with type 2 diabetes

**DOI:** 10.1186/s12902-023-01440-0

**Published:** 2023-09-04

**Authors:** Luyan Zhang, Qian Guo, Yanjia Xu, Wenzhen Wei, Yu Wang

**Affiliations:** 1 Department of Geriatric Endocrinology, The People’s Hospital of Gansu Province, Lanzhou, 730000 Gansu Province China; 2 Yueguang, Asia-Pacific, Duanjiatan, East Section of Donggang Road, Chengguan District, Lanzhou City, Gansu Province China

**Keywords:** 25- hydroxyvitamin D, Albuminuiria, Diabetes

## Abstract

**Objective:**

The objective was to analyze the relationship between serum 25-hydroxy-vitamin D (25(OH)D) level and albuminuiria in middle-aged and older patients with type 2 diabetes of Gansu Province.

**Methods:**

Data pertaining to 380 in-patients with type 2 diabetes were collected. Subjects were classified groups based on gender,age,25(OH)D,BMI and UACR.Serum 25(OH)D and other clinical characteristics among various UACR groups were compared.The relationship between albuminuiria and 25(OH)D was analyzed.

**Results:**

Out of the 380 subjects, 83.4%were classified as vitamin D deficiency, 14.5%were classified as vitamin D insufficiency, while 2.1% were classified as vitamin D sufficiency. Among the participants,41% had albuminuria (microalbuminuria,28.7%;macroalbuminuria,12.3%).The prevalence of 25(OH)D deficiency in the albuminuria group(84.6%) was significantly higher than that in the normoalbuminuria group(82.6%)(Mann–Whitney U test:Z = -3.86,*P* = 0.000); patients with macroalbuminuria had the highest prevalence of 25(OH)D deficiency (91.5%; *P* < 0.01 versus normoalbuminuria).A binary logistic analysis demonstrated that 25(OH)D were protective factors for albuminuria.

**Conclusions:**

The prevalence of vitamin D deficiency in patients with albuminuria was overtly higher than that in patients without albuminuria among middle-aged and older adults with type 2 diabetes.

**Supplementary Information:**

The online version contains supplementary material available at 10.1186/s12902-023-01440-0.

## Introduction

The accelerated aging of the population has led to an increase in the number of older patients with type 2 diabetes mellitus (T2DM) [1]. Diabetic kidney disease (DKD) is one of the main consequences and a leading cause of death in people with type 2 diabetes, and its prevalence is rising globally as more middle-aged and older patients get the condition [2]. DKD has a significant impact on patients' quality of life and safety because it is the leading cause of disability and death in middle-aged and older people with type 2 diabetes [3]. Progressive albuminuria and a deterioration in renal function are the main clinical signs of DKD [4, 5]. DKD is characterized by podocyte destruction, interstitial fibrosis, and thickening of the glomerular basement membrane [6].The level of vitamin D in diabetic patients is lower than that in the normal population [7].Vitamin D deficiency may be involved in the occurrence and development of diabetic nephropathy [8–10]. A form of fat-soluble vitamin called serum vitamin D is crucial for maintaining bone health as it helps control the metabolism of calcium and phosphate [11]. In addition to causing problems with the way calcium and phosphorus are metabolized and with how bones are mineralized, a lack of vitamin D also hastens the onset of chronic renal disease [12].Numerous observational studies showed that patients with type 2 diabetes in Gansu Province, whether they had albuminuria or not, had vitamin D insufficiency or deficiency [13, 14]. Recent evidence from human and animal studies suggests that chronic kidney disease's traditional symptoms include low vitamin D levels and vitamin D resistance [15, 16]. Widespread use of serum 25-hydroxyvitamin D (25(OH)D) concentration as a diagnostic for vitamin D status [17]. Meanwhile,studies have confirmed that vitamin D deficiency is common in the adult population of Gansu Province [18].

Therefore, this study aims to evaluate the relationship between serum 25(OH)D and albuminuria in middle-aged and older patients with type 2 diabetes with or without albuminuiria of Gansu Province. Our findings may offer proof for the management and prevention of DKD in this area.

## Objects and methods

### Research objects

A total of 380 patients with type 2 diabetes (283 males and 97 females) who were hospitalized in the Department of Cadre Endocrinology of The People's Hospital of Gansu Province from October 2019 to December 2021. This cross-sectional research was carried out with an average age of (65.09 ± 8.20) years (55-92years).

(1) The following were the selection criteria: an endocrinologist determined the presence of type 2 diabetes mellitus based on the patient's medical history and high blood sugar levels. The following qualifications were required for exclusion:hepatorenal syndrome, hyperparathyroidism, malignant tumor, chronic digestive system disease, cancer, other special types of diabetes, acute complications of diabetes, inflammatory reaction, infection and recent surgery, estimated glomerular filtration rate (GFR) < 60mL/min,and vitamin D before hospitalization was excluded patients on complementary therapy. Our study protocol was approved by the ethics committee of hospital medicine. Written informed consent was obtained from each participant before data collection.

### Research methods

#### General information

Collect general information of all patients, including name, age, gender, height, body mass index (BMI) = weight (kg)/height (m)^2^.

#### Laboratory measurements

Blood was drawn from the subjects fasting for 8–12 h,a random spot urine sample was collected. Fasting blood glucose (FBG), lipid profile, blood urea nitrogen (BUN), serum creatinine (Scr), calcium, phosphate, and HbA1c were among the laboratory markers. Using an automated analyzer called the Abbott C-1600, blood biochemical indicators were looked at.Using the glucose oxidase technique, FBG was found.Urinary albumin and creatinine concentrations were measured using a turbidimetric immunoassay with a Hitachi-7600 analyzer. Albuminuria was defined according to the creatinine,which was calculated by dividing the urinary albumin concentration (mg) by the urinary creatinine concentration (mg), which was urinary albumin-to-creatinine ratio (UACR).We determined serum 25(OH)D concentrations using using immunoassay chemiluminescence detection on Abbott ISR-2000 automatic analyzer.HbA1c was measured by ionic exchange high-performance liquid chromatography. Data support was provided by the Laboratory Department of The People's Hospital of Gansu Province.

#### Diagnostic categories

Categories was defined as follows.Based on the gender:283 males and 97 females.

Vitamin D status was divided into the following three categories based on serum 25(OH)D levels, as per the diagnostic criteria in the Application Guidelines for Vitamin D [19]: vitamin D sufficiency (> 30μg/L)(8); vitamin D insufficiency (> 20, ≤ 30μg/L)(55); and vitamin D deficiency(≤ 20μg/L)(317).Based on the levels of the level of urinary albumin-to-creatinine ratio (UACR),subjects were classified into three groups:UACR levels of 30 mg/g or less were classified as normoalbuminuria(224), whereas those of 30-300mg/g(≧30mg/g,and < 300mg/g)and above 300mg/g were classified as microalbuminuria(109) and macroalbuminuria(47), respectively. Based on their chronological ages, subjects were classified into three groups:young aged 55–64(204),middle-aged 65–74(120) and older over 75 years(56). Different UACR groups' serum 25(OH)D levels and other clinical traits were compared.Analysis was done on the connection between albuminuria and 25(OH)D.

### Statistical analysis

The SPSS version 26.0 software utilized to carry out all statistical calculations. Continuous data with normal distribution are provided as mean standard deviation (SD), while continuous variables with non-normal distribution are shown as median interquartile range (IQR).The presentation of categorical variables is as frequency(percentage). For continuous variables with a non-normal distribution, between-group differences were evaluated using the Mann–Whitney U test.The correlation between the indicators was examined using Spearman correlation.Using binary logistic analysis, after taking into account potential confounding factors, the odds ratios (OR) and associated 95% confidence intervals (CI) for microalbuminuria and macroalbuminuria in connection to vitamin D status were determined. Two-sided *P* values of 0.05 or less were taken to indicate statistical significance across all analyses.

## Results

### General characteristics

Two hundred eighty-three (74.5%) of the 380 individuals were men, while 97 (25.5%) were women. Because more diabetic women were disqualified because of their propensity for urinary tract infections, which affect urine protein excretion, the percentage of females was lower. Out of the 380 subjects, the overall 25(OH)D level was 12.9 (9.9, 17.0)μg/L, patients had a considerably higher ratio of vitamin D deficiency and insufficiency(97.9%).

Among them, 317 (83.4%) were classified as vitamin D deficiency [25(OH)D ≤ 20 μg/L], 55 (14.5%) were classified as vitamin D insufficiency[25(OH)D > 20, ≤ 30 μg/L], while 8 (2.1%) were classified as vitamin D sufficiency [25(OH)D > 30 μg/L]. According to HbA1c levels, among the patients, 94 (24.7%) had HbA1c values below 7%, 213 (56.1%) had levels between 7 and 10%, and 73 (19.2%) had levels beyond 10%.

### Clinical traits across different age groups compared:

The patients were divided into three groups according to age: young aged 55–64 years(204),middle-aged 65–74 years(120) and older over 75 years(56). Mann–Whitney U test results: there were significant differences in UACR between the middle-aged and the older aged group, *p* < 0.05.The test also showed that significant differences were observed between the young aged and the older aged group with respect to UACR and 25(OH)D, *p* < 0.05. (see Table 1).

**Table 1:**
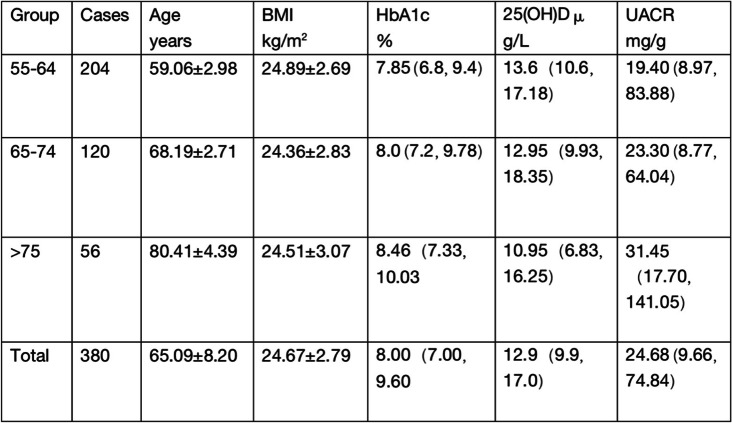
Information of patients with type 2 diabetes by age [Mean ± SD, median (IQR)]

*BMI* body mass index, *HbA1c* glycosylated hemoglobin, *25(OH)D* 25-hydroxyvitamin D, *UACR* urinary albumin-to-creatinine ratio

### Comparison of clinical characteristics among various UACR groups

Among the participants,156(41%) had albuminuria (microalbuminuria,28.7%;macroalbuminuria,12.3%). The prevalence of 25(OH)D deficiency in the albuminuria group(84.6%) was significantly higher than that in the normoalbuminuria group(82.6%)(Mann–Whitney U test:Z = -3.86,*P* = 0.000); the prevalence of 25(OH)D deficit was highest in patients with macroalbuminuria(91.5%; *P* < 0.01 versus normoalbuminuria). The Mann–Whitney U test showed that strong differences were found between the groups with macroalbuminuria and normoalbuminuria groups with respect to 25(OH)D,*p* < 0.001. Compared with the microalbuminuria and macroalbuminuria groups, the difference in 25(OH)D was statistically significant,*p* < 0.05. The differences in 25(OH)D between the microalbuminuria and the normoalbuminuria groups were statistically significant (*p* < 0.05). (see Table 2).

**Table 2:**
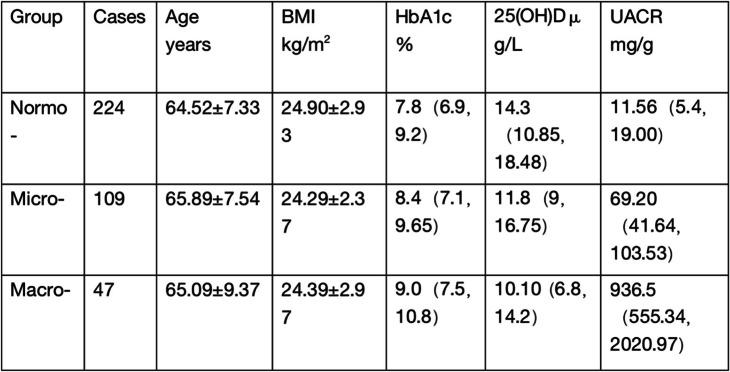
Information of patients with type 2 diabetes grouped by albuminuria [Mean ± SD, median (IQR)]

*Normo* normoalbuminuria, *Micro* microalbuminuria, *Macro* macroalbuminuria

## Correlation comparison

### Spearman correlation analysis between 25(OH)D and albuminuiria in middle-aged and older diabetic patients

The studies that demonstrated a negative and substantial correlation between vitamin D and UACR(*r* = -0.233, *p* < 0.001), HbA1c(*r* = -0.223, *p* < 0.001).According to different age groups, in the young aged group(55-64years) and the middle-aged group(65-74years), 25(OH)D levels were negatively correlated with UACR(*p* < 0.05), and the middle-aged group was more significant (*p* < 0.001) (see Table 3).

**Table 3:**
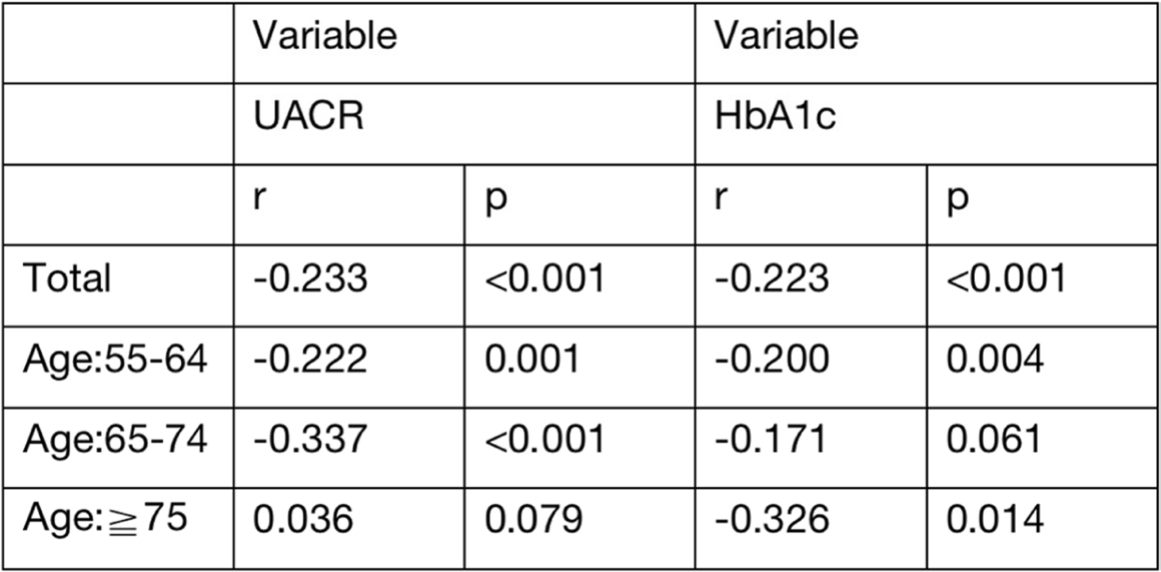
Spearman correlation analysis of 25(OH)D with albuminuiria in T2DM patients

## Analysis of the risk factors for albuminuria in type 2 diabetes using binary logistic regression

With or without albuminuria as the dependent variable [without albuminuria (UACR < 30mg/g) = 0, with albuminuria(UACR≧30mg/g) = 1],and 25(OH)D as an independent variables(assigned value: measured value).The results showed that 25(OH)D was protective factor for albuminuria (See Table 4).

**Table 4:**
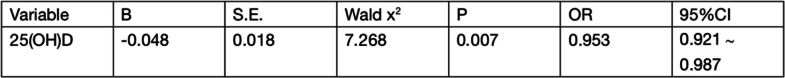
Binary logistic regression analysis of risk factors for albuminuria with T2DM

## Discussion

We investigated the association of 25(OH)D with albuminuiria in the type 2 diabetes aged≧55y.In this observational study, we found that a low plasma 25(OH)D level was associated with a higher odds of having increased albuminuria.

The results of this study showed the middle-aged and older patients with type 2 diabetes who were treated in our hospital had obvious vitamin D deficiency, and the deficiency rate was 83.4%, which was similar to the proportion of vitamin D deficiency in the middle-aged and older people in Gansu (81.9%) previously reported [19]. These results showed that type 2 diabetes patients in China frequently have vitamin D deficiency or insufficiency. A large number of epidemiological studies at home and abroad [11] show that vitamin D shortage or insufficiency is relatively frequent throughout the world;with a deficiency rate of 39.6% to 57%, among which the older account for the majority [20]. It has been recently described in the literature that vitamin D has a strong protective role in chronic kidney disease [16, 17, 21]. One possible mechanism is that vitamin D protects against the development of DKD by inhibiting the activation of RAS by reducing renin expression [22–24]. In addition, 25(OH)D may act lowering oxidative stress by improving renal antioxidant capacity, preventing damage to podocytes by inhibiting hyperglycemia-induced apoptosis, promoting anti-inflammatory action, and improving endothelial function [15, 25, 26]. Kuhlmann et al. [27] found an animal study that vitamin D prevents podocyte damage, which lowers proteinuria and glomerulosclerosis. Meanwhile, another study showed that that lower 25(OH)D were linked to a higher prevalence of albuminuria in a sizable representative sample of adults with a variety of age, racial/ethnic, and diabetes status [28] (population). The conclusion agreed with what we found in the study. Regardless of the processes behind the link between a lack of vitamin D and albuminuria,numerous evidences point to vitamin D's major role in the prevalence of albuminuria and the existence of diabetic nephropathy [29].

In addition, we found that the serum 25(OH)D level of T2DM patients with albuminuiria was significantly lower than that of patients without albuminuiria. This study also showed that patients with micro or macro albuminuria had a higher prevalence of vitamin D insufficiency or deficiency compared to those with normoalbuminuria,and the difference between the groups was statistically significant.Our results agree with Ucak et al. [30], who discovered a link between individuals with low 25(OH)D levels and a higher incidence of microalbuminuria.

Further,elevated UACR level was associated with decreased 25(OH)D. Our study was also confirmed that vitamin D deficiency was more serious in older patients with type 2 diabetic nephropathy. The results confirmed that the level of 25(OH)D in patients aged 65–74 was more significantly correlated with UACR. Partial correlation (spearman) analysis showed that UACR level was significantly negatively correlated with 25(OH)D.In accordance with the our current study, binary logistic regression analysis found that 25(OH)D level was correlated with albuminuria.

Our investigation has the advantage of include a large sample of patients while eliminating those who had clearly compromised renal function (high serum creatinine levels), which would have interfered with 25(OH)D metabolism.Some limitations of our study should be acknowledged.Firstly,the lack of a control group of the same age and the influence of oral hypoglycemic drugs on bone metabolism are not excluded. Secondly, given that this was a cross-sectional study, the design makes it difficult to understand the causal relationship between serum vitamin D and proteinuria, which is a limitation of this research.The low vit D can give rise to albuminuria as easily as albuminuria can give low vitamin D,in compliance with albuminuria can give rise to low vitamin D carrier protein (alpha globulin) [ the protein is lost in the urine] hat in turn lowers totalvit D levels [31]. Finally, the impact of unmeasured confounders, such as exposure to direct sunlight, nutritional status, and smoking status, could not completely be ruled out even after we had controlled for several potential confounding factors.Therefore, more research and prospective cohort studies are needed to determine the exact cause of the link between vitamin D and protainuria.

In conclusion, we found that the low plasma 25(OH)D level was associated with a higher odds of having increased albuminuria.Vitamin D deficiency has become a global public health problem, especially for middle-aged and older patients, which is an important issue related to the future of mankind.

Therefore, vitamin D level monitoring should be carried out in patients with type 2 diabetes with or without albuminuiria, which not only helps to identify high-risk groups of diabetic nephropathy, but also provides a reference for judging the severity of diabetic nephropathy. In addition to establishing a 25(OH)D reference range specifically for middle-aged and older Chinese patients, more studies with adequate serum vitamin D are required, including randomized clinical trials, for a more forceful clinical practice among this population. Supplement the clinical value of vitamin D in preventing the occurrence of diabetes and preventing the progression of diabetic nephropathy is still worthy of further exploration.

### Supplementary Information

**Additional file 1. **

## Data Availability

All data generated or analyzed during this study are included in this published article and its supplementary information files.
