# Supplementary figures and images for: Association between serum 25- hydroxyvitamin D and albuminuiria in middle-aged and older Chinese patients with type 2 diabetes
Source: BMC Endocr Disord. 2023 Sep 4;23:189. doi: 10.1186/s12902-023-01440-0 (PMC10476337; doi:10.1186/s12902-023-01440-0)

Supplementary file 1


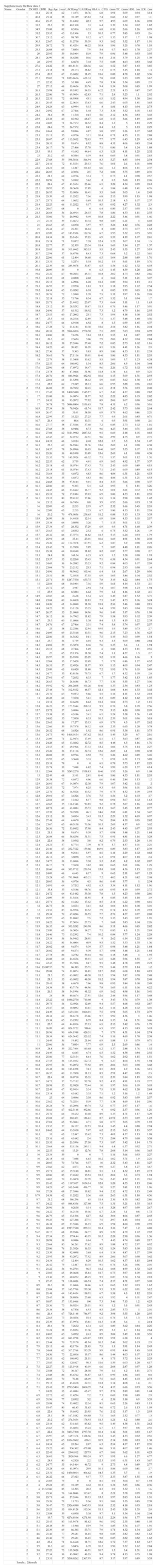

Supplement: Supplementary file 1 — Additional file 1. [file 12902_2023_1440_MOESM1_ESM.docx]
